# How often do follow-on activities occur - trends seen in a patent database for GPCRs

**DOI:** 10.1186/1758-2946-4-S1-P51

**Published:** 2012-05-01

**Authors:** Christian Tyrchan, Sorel Muresan

**Affiliations:** 1 AstraZeneca R&D, CVGI iMed, Pepparedsleden 1, S-43183 Mölndal, Sweden; 2 AstraZeneca R&D, DECS, Pepparedsleden 1, S-43183 Mölndal, Sweden

## 

The first compound reaching the market following the approval of the official offices is referred to as first-in-class or breakthrough drug. These compounds constitute a new class of drugs meaning that they introduce a novel mode of action (MoA) or provide a significant improvement over the standard therapy, if not enables a therapy, in terms of efficacy and safety. A follow-on (or me-too) drug is, in the common understanding, a chemical entity which has structural similarity or has the same pharmacological MoA as the first-in-class drug. The approach of follow-on drugs is controversially discussed in the literature [1-3].

Based on the GVKBIO Medicinal Chemistry and Target Class databases (which capture explicit relationships between published documents, compounds, assay results and targets), we investigated the occurrence of drug discovery follow-on activities as captured by the pharmaceutical patent space [4]. To do so, we abstracted from the GVKBIO databases 11,827 patents which are linked to a GPCR target with a defined Entrez Gene ID (as November 2010). In a next step, we removed all peptide structures because of the size and the similarity of their backbone. Subsequently, we kept all 10,253 patents belonging to the TOP 100 companies in terms of number of patents published. The set was consolidated by merging patents for known mergers and acquisitions until 2008. All possible patent combinations were created if they share an official gene name and are published by different companies within a 6-year interval. Similarity descriptors and scores could be generated for 1,570,381 out of a total of 1,608,368 pairs (97.6%) [5].

As expected, the pharmaceutical research is highly competitive and dynamic. This is supported by the fact that most of the follow-on patents are published within the first two years with a high number of patent pairs published in the same year. Around 4800 (47%) patents included in our analysis are linked to follow-on activities with only small differences between the companies.
